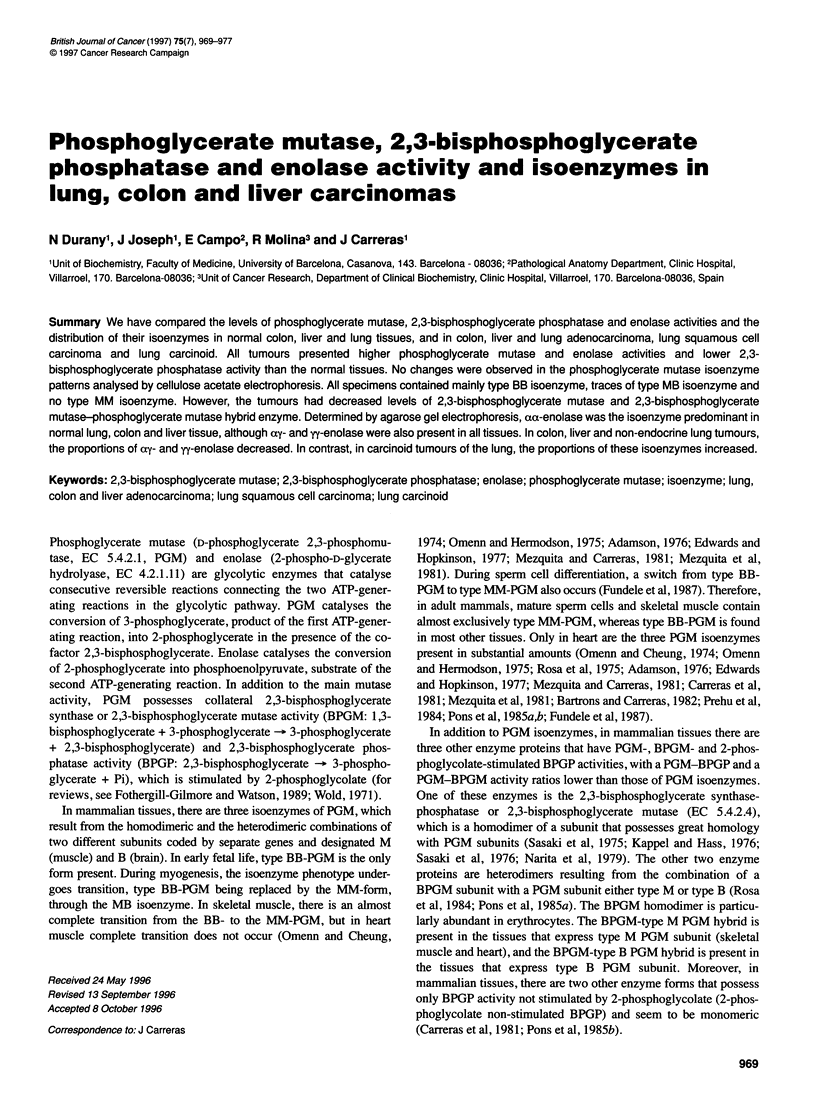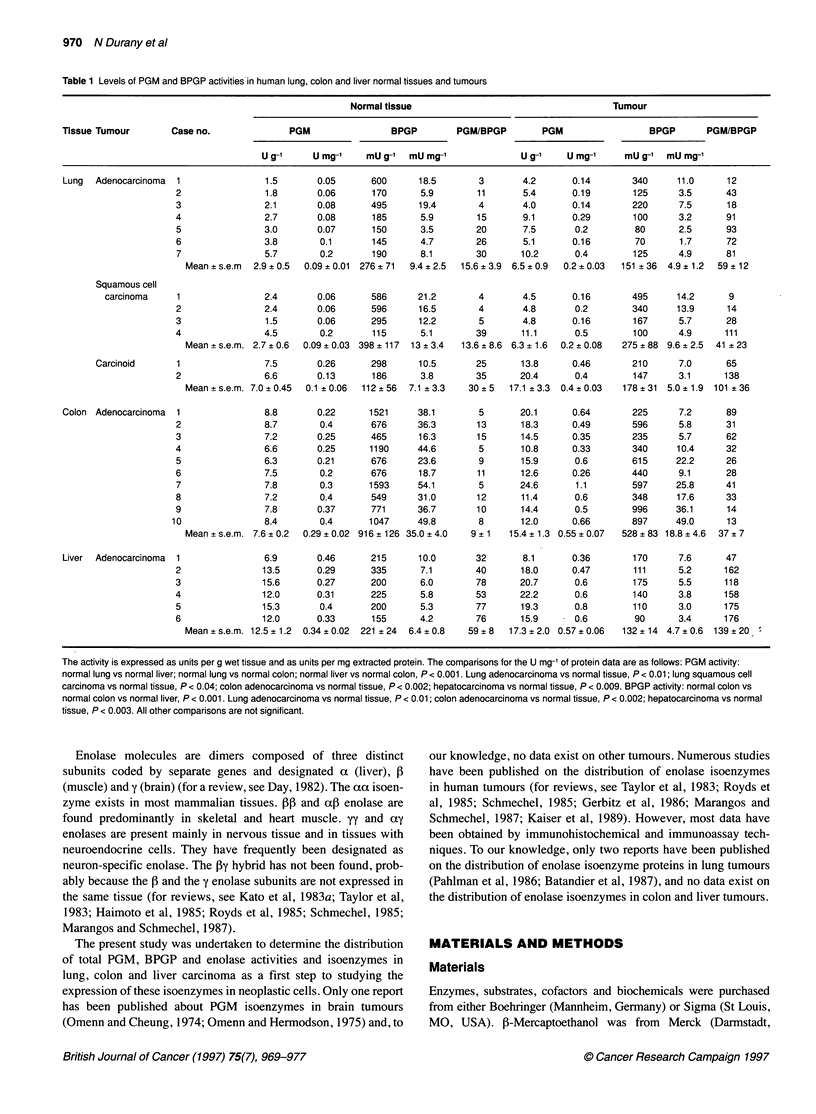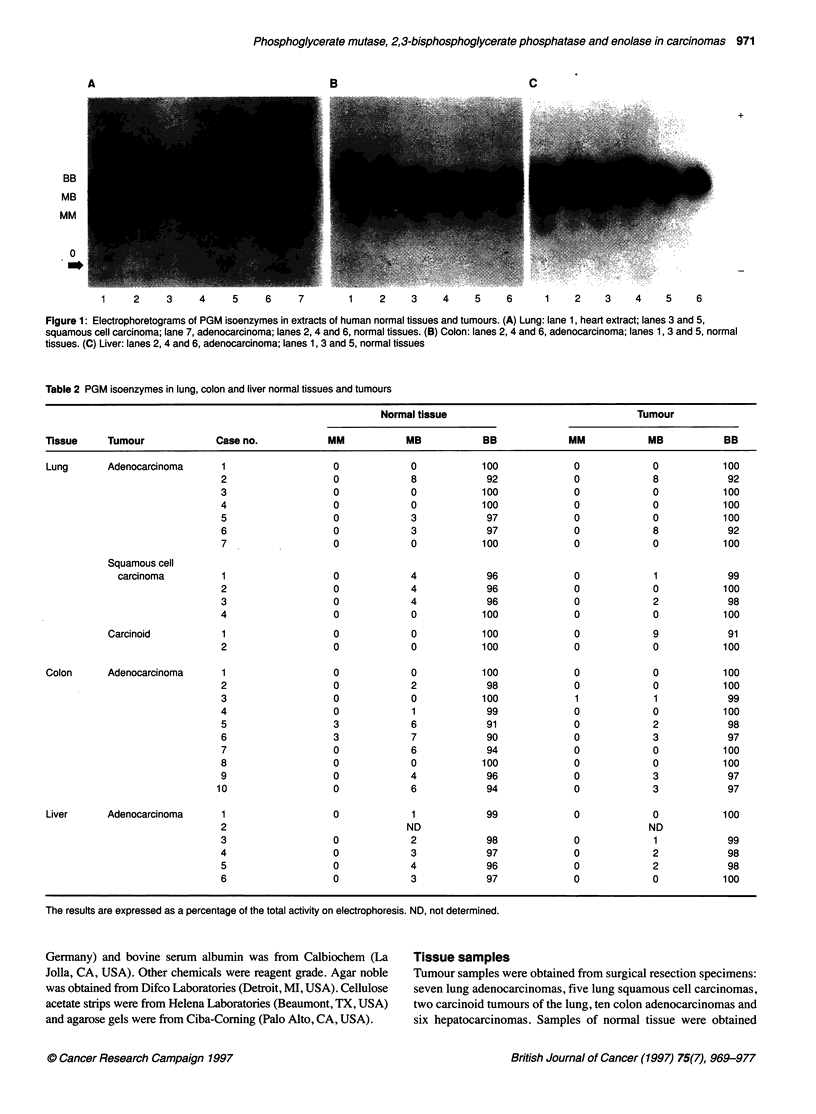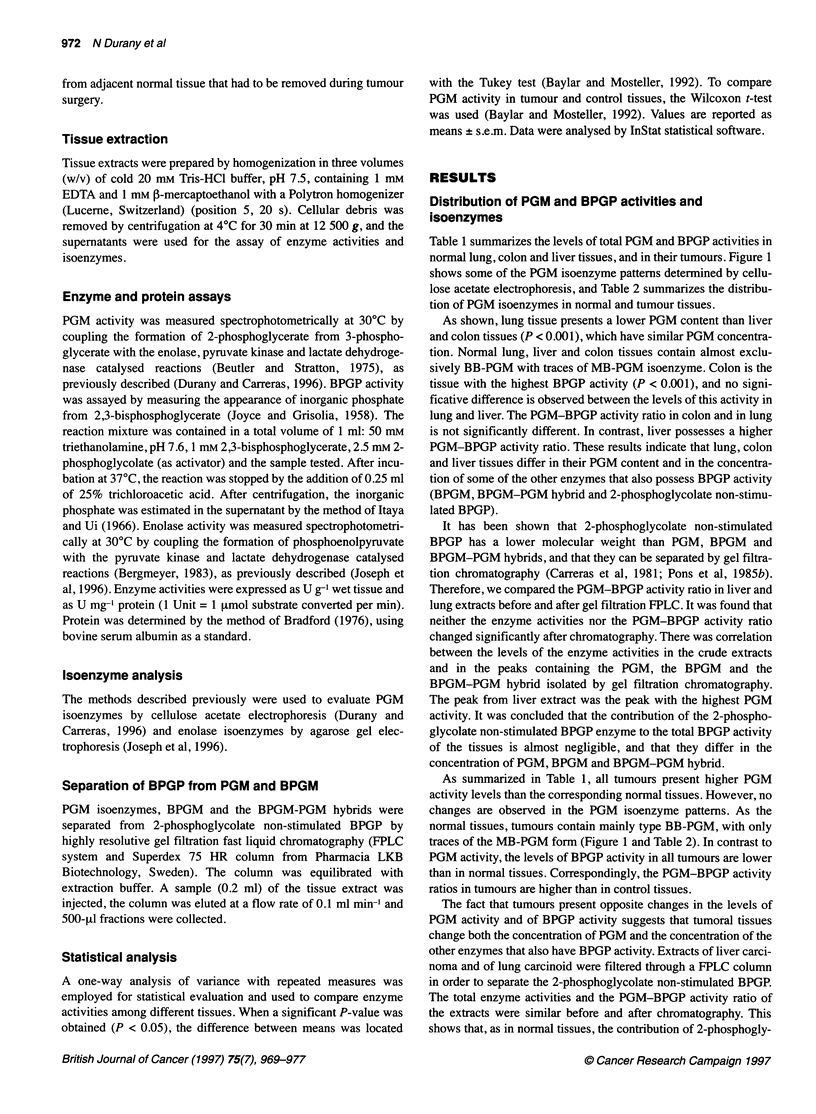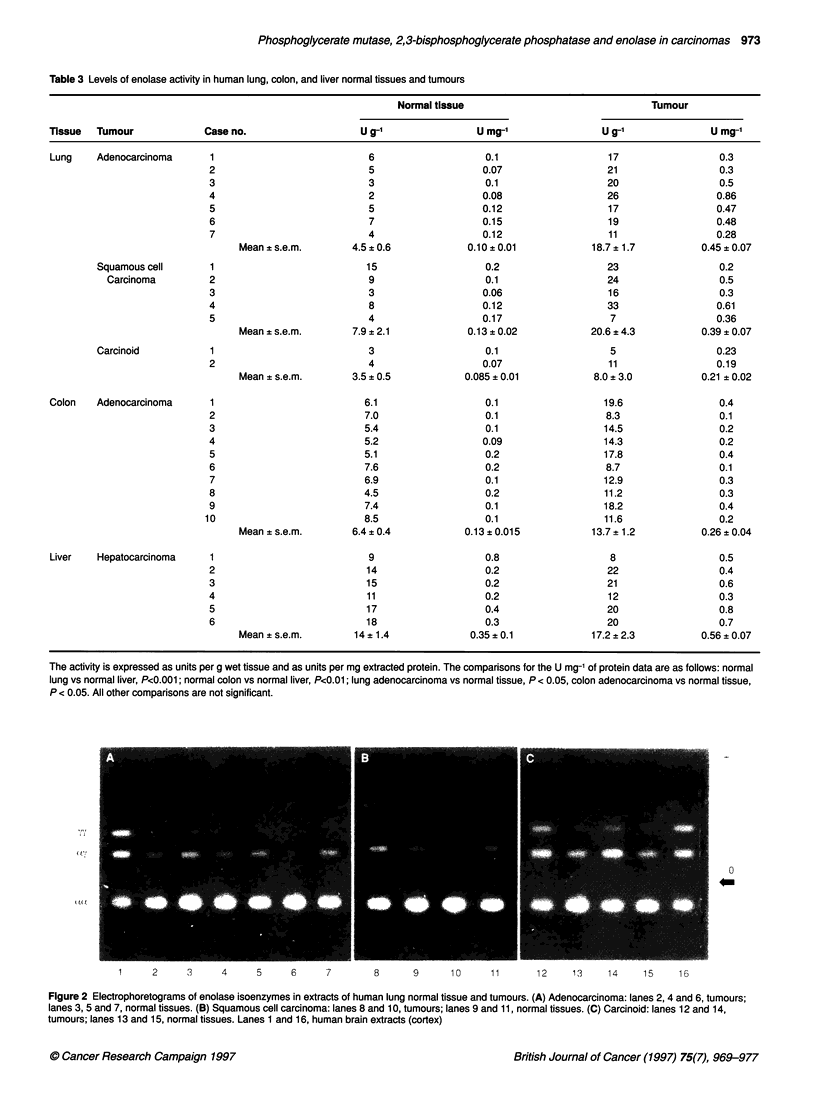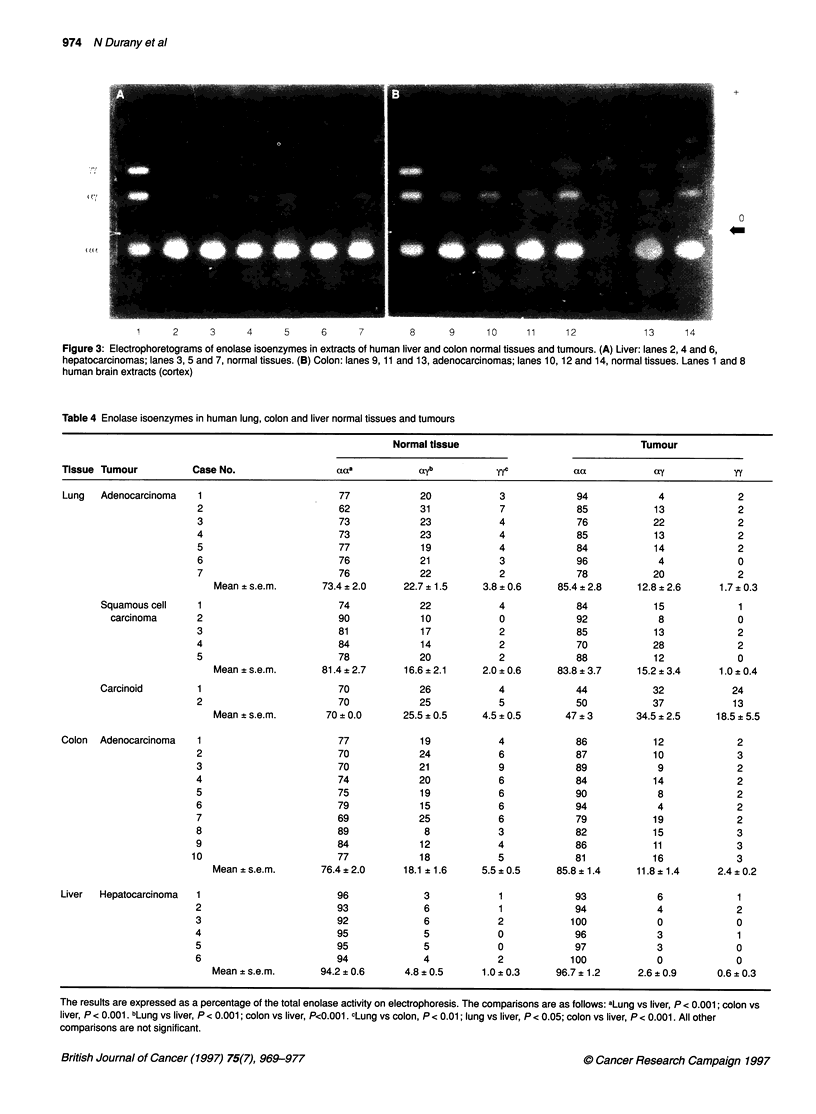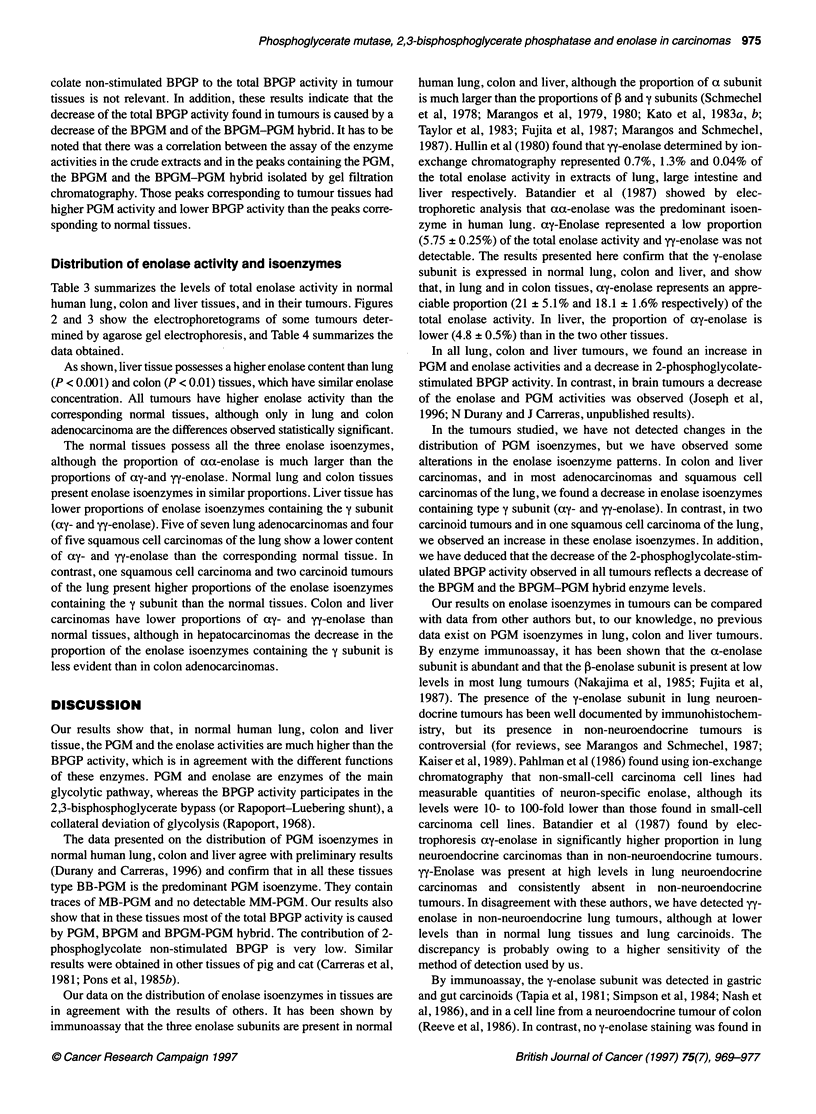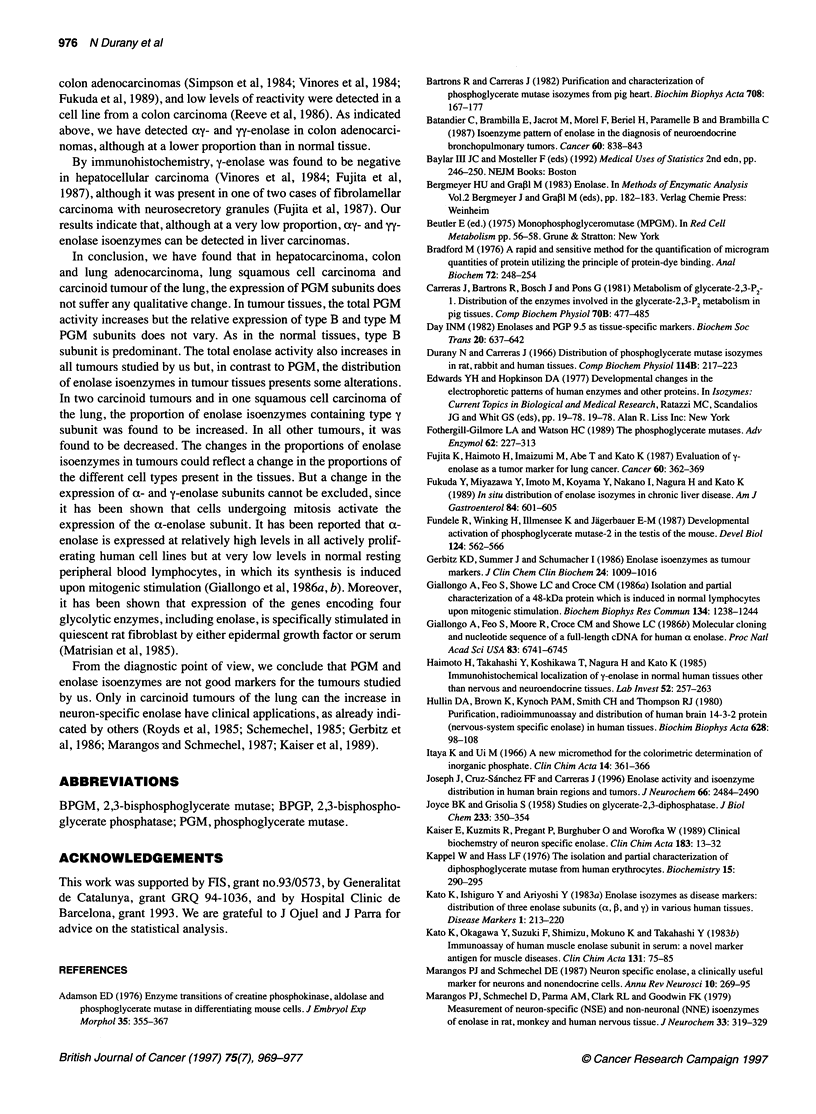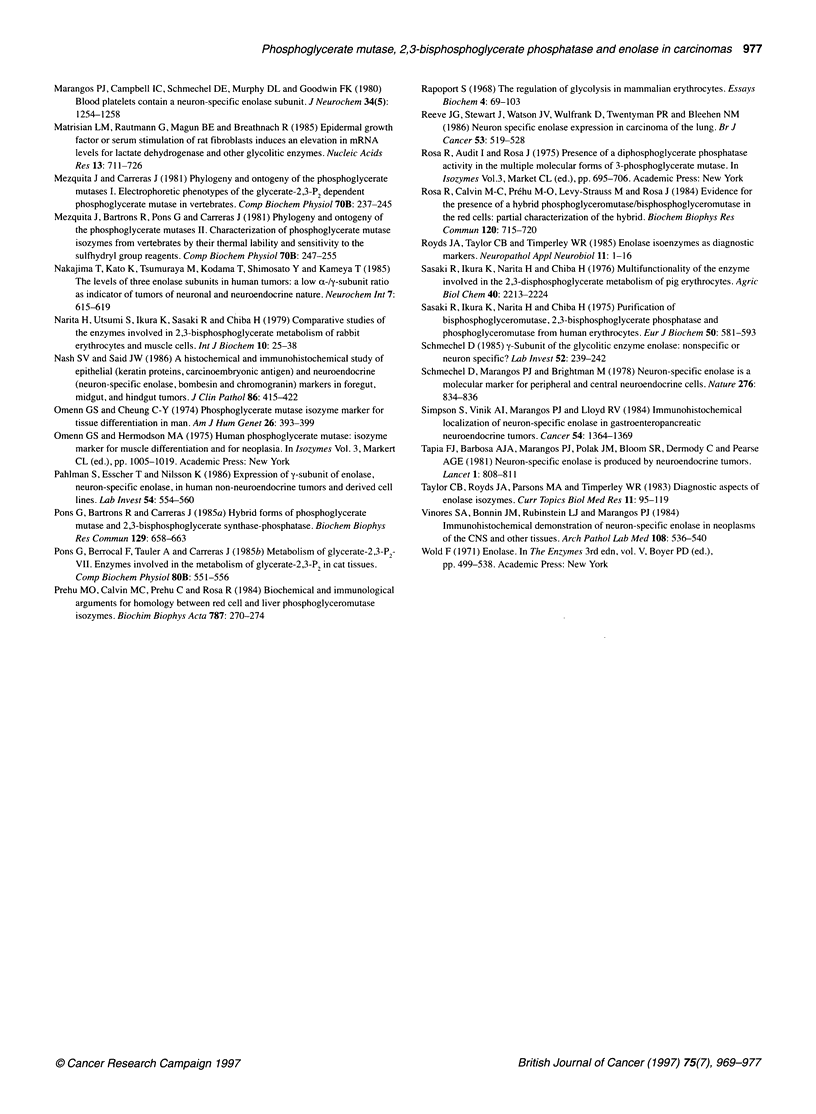# Phosphoglycerate mutase, 2,3-bisphosphoglycerate phosphatase and enolase activity and isoenzymes in lung, colon and liver carcinomas.

**DOI:** 10.1038/bjc.1997.168

**Published:** 1997

**Authors:** N. Durany, J. Joseph, E. Campo, R. Molina, J. Carreras

**Affiliations:** Unit of Biochemistry, Faculty of Medicine, University of Barcelona, Casanova, Spain.

## Abstract

**Images:**


British Journal of Cancer (1997) 75(7), 969–977
© 1997 Cancer Research Campaign

### Phosphoglycerate mutase, 2,3-bisphosphoglycerate phosphatase and enolase activity and isoenzymes in lung, colon and liver carcinomas

**N Durany[1], J Joseph[1], E Campo[2], R Molina[3] and J Carreras[1]**

[1]Unit of Biochemistry, Faculty of Medicine, University of Barcelona, Casanova, 143. Barcelona - 08036; [2]Pathological Anatomy Department, Clinic Hospital, Villarroel, 170. Barcelona-08036; [3]Unit of Cancer Research, Department of Clinical Biochemistry, Clinic Hospital, Villarroel, 170. Barcelona-08036, Spain

**Summary** We have compared the levels of phosphoglycerate mutase, 2,3-bisphosphoglycerate phosphatase and enolase activities and the distribution of their isoenzymes in normal colon, liver and lung tissues, and in colon, liver and lung adenocarcinoma, lung squamous cell carcinoma and lung carcinoid. All tumours presented higher phosphoglycerate mutase and enolase activities and lower 2,3-bisphosphoglycerate phosphatase activity than the normal tissues. No changes were observed in the phosphoglycerate mutase isoenzyme patterns analysed by cellulose acetate electrophoresis. All specimens contained mainly type BB isoenzyme, traces of type MB isoenzyme and no type MM isoenzyme. However, the tumours had decreased levels of 2,3-bisphosphoglycerate mutase and 2,3-bisphosphoglycerate mutase–phosphoglycerate mutase hybrid enzyme. Determined by agarose gel electrophoresis, αα-enolase was the isoenzyme predominant in normal lung, colon and liver tissue, although αγ- and γγ-enolase were also present in all tissues. In colon, liver and non-endocrine lung tumours, the proportions of αγ- and γγ-enolase decreased. In contrast, in carcinoid tumours of the lung, the proportions of these isoenzymes increased.

**Keywords:** 2,3-bisphosphoglycerate mutase; 2,3-bisphosphoglycerate phosphatase; enolase; phosphoglycerate mutase; isoenzyme; lung, colon and liver adenocarcinoma; lung squamous cell carcinoma; lung carcinoid

Phosphoglycerate mutase (D-phosphoglycerate 2,3-phosphomutase, EC 5.4.2.1, PGM) and enolase (2-phospho-D-glycerate hydrolyase, EC 4.2.1.11) are glycolytic enzymes that catalyse consecutive reversible reactions connecting the two ATP-generating reactions in the glycolytic pathway. PGM catalyses the conversion of 3-phosphoglycerate, product of the first ATP-generating reaction, into 2-phosphoglycerate in the presence of the cofactor 2,3-bisphosphoglycerate. Enolase catalyses the conversion of 2-phosphoglycerate into phosphoenolpyruvate, substrate of the second ATP-generating reaction. In addition to the main mutase activity, PGM possesses collateral 2,3-bisphosphoglycerate synthase or 2,3-bisphosphoglycerate mutase activity (BPGM: 1,3-bisphosphoglycerate + 3-phosphoglycerate → 3-phosphoglycerate + 2,3-bisphosphoglycerate) and 2,3-bisphosphoglycerate phosphatase activity (BPGP: 2,3-bisphosphoglycerate → 3-phosphoglycerate + Pi), which is stimulated by 2-phosphoglycolate (for reviews, see Fothergill-Gilmore and Watson, 1989; Wold, 1971).

In mammalian tissues, there are three isoenzymes of PGM, which result from the homodimeric and the heterodimeric combinations of two different subunits coded by separate genes and designated M (muscle) and B (brain). In early fetal life, type BB-PGM is the only form present. During myogenesis, the isoenzyme phenotype undergoes transition, type BB-PGM being replaced by the MM-form, through the MB isoenzyme. In skeletal muscle, there is an almost complete transition from the BB- to the MM-PGM, but in heart muscle complete transition does not occur (Omenn and Cheung, 1974; Omenn and Hermodson, 1975; Adamson, 1976; Edwards and Hopkinson, 1977; Mezquita and Carreras, 1981; Mezquita et al, 1981). During sperm cell differentiation, a switch from type BB-PGM to type MM-PGM also occurs (Fundele et al, 1987). Therefore, in adult mammals, mature sperm cells and skeletal muscle contain almost exclusively type MM-PGM, whereas type BB-PGM is found in most other tissues. Only in heart are the three PGM isoenzymes present in substantial amounts (Omenn and Cheung, 1974; Omenn and Hermodson, 1975; Rosa et al, 1975; Adamson, 1976; Edwards and Hopkinson, 1977; Mezquita and Carreras, 1981; Carreras et al, 1981; Mezquita et al, 1981; Bartrons and Carreras, 1982; Prehu et al, 1984; Pons et al, 1985*a,b*; Fundele et al, 1987).

In addition to PGM isoenzymes, in mammalian tissues there are three other enzyme proteins that have PGM-, BPGM- and 2-phosphoglycolate-stimulated BPGP activities, with a PGM–BPGP and a PGM–BPGM activity ratios lower than those of PGM isoenzymes. One of these enzymes is the 2,3-bisphosphoglycerate synthase-phosphatase or 2,3-bisphosphoglycerate mutase (EC 5.4.2.4), which is a homodimer of a subunit that possesses great homology with PGM subunits (Sasaki et al, 1975; Kappel and Hass, 1976; Sasaki et al, 1976; Narita et al, 1979). The other two enzyme proteins are heterodimers resulting from the combination of a BPGM subunit with a PGM subunit either type M or type B (Rosa et al, 1984; Pons et al, 1985*a*). The BPGM homodimer is particularly abundant in erythrocytes. The BPGM-type M PGM hybrid is present in the tissues that express type M PGM subunit (skeletal muscle and heart), and the BPGM-type B PGM hybrid is present in the tissues that express type B PGM subunit. Moreover, in mammalian tissues, there are two other enzyme forms that possess only BPGP activity not stimulated by 2-phosphoglycolate (2-phosphoglycolate non-stimulated BPGP) and seem to be monomeric (Carreras et al, 1981; Pons et al, 1985*b*).

*Received 24 May 1996*
*Revised 13 September 1996*
*Accepted 8 October 1996*

*Correspondence to:* J Carreras

**Table 1** Levels of PGM and BPGP activities in human lung, colon and liver normal tissues and tumours

| | | | Normal tissue | | | | | Tumour | | | | |
|---|---|---|---|---|---|---|---|---|---|---|---|---|
| Tissue | Tumour | Case no. | PGM | | BPGP | | PGM/BPGP | PGM | | BPGP | | PGM/BPGP |
| | | | U $g^{-1}$ | U $mg^{-1}$ | mU $g^{-1}$ | mU $mg^{-1}$ | | U $g^{-1}$ | U $mg^{-1}$ | mU $g^{-1}$ | mU $mg^{-1}$ | |
| Lung | Adenocarcinoma | 1 | 1.5 | 0.05 | 600 | 18.5 | 3 | 4.2 | 0.14 | 340 | 11.0 | 12 |
| | | 2 | 1.8 | 0.06 | 170 | 5.9 | 11 | 5.4 | 0.19 | 125 | 3.5 | 43 |
| | | 3 | 2.1 | 0.08 | 495 | 19.4 | 4 | 4.0 | 0.14 | 220 | 7.5 | 18 |
| | | 4 | 2.7 | 0.08 | 185 | 5.9 | 15 | 9.1 | 0.29 | 100 | 3.2 | 91 |
| | | 5 | 3.0 | 0.07 | 150 | 3.5 | 20 | 7.5 | 0.2 | 80 | 2.5 | 93 |
| | | 6 | 3.8 | 0.1 | 145 | 4.7 | 26 | 5.1 | 0.16 | 70 | 1.7 | 72 |
| | | 7 | 5.7 | 0.2 | 190 | 8.1 | 30 | 10.2 | 0.4 | 125 | 4.9 | 81 |
| | | Mean ± s.e.m | 2.9 ± 0.5 | 0.09 ± 0.01 | 276 ± 71 | 9.4 ± 2.5 | 15.6 ± 3.9 | 6.5 ± 0.9 | 0.2 ± 0.03 | 151 ± 36 | 4.9 ± 1.2 | 59 ± 12 |
| | Squamous cell carcinoma | 1 | 2.4 | 0.06 | 586 | 21.2 | 4 | 4.5 | 0.16 | 495 | 14.2 | 9 |
| | | 2 | 2.4 | 0.06 | 596 | 16.5 | 4 | 4.8 | 0.2 | 340 | 13.9 | 14 |
| | | 3 | 1.5 | 0.06 | 295 | 12.2 | 5 | 4.8 | 0.16 | 167 | 5.7 | 28 |
| | | 4 | 4.5 | 0.2 | 115 | 5.1 | 39 | 11.1 | 0.5 | 100 | 4.9 | 111 |
| | | Mean ± s.e.m. | 2.7 ± 0.6 | 0.09 ± 0.03 | 398 ± 117 | 13 ± 3.4 | 13.6 ± 8.6 | 6.3 ± 1.6 | 0.2 ± 0.08 | 275 ± 88 | 9.6 ± 2.5 | 41 ± 23 |
| | Carcinoid | 1 | 7.5 | 0.26 | 298 | 10.5 | 25 | 13.8 | 0.46 | 210 | 7.0 | 65 |
| | | 2 | 6.6 | 0.13 | 186 | 3.8 | 35 | 20.4 | 0.4 | 147 | 3.1 | 138 |
| | | Mean ± s.e.m. | 7.0 ± 0.45 | 0.1 ± 0.06 | 112 ± 56 | 7.1 ± 3.3 | 30 ± 5 | 17.1 ± 3.3 | 0.4 ± 0.03 | 178 ± 31 | 5.0 ± 1.9 | 101 ± 36 |
| Colon | Adenocarcinoma | 1 | 8.8 | 0.22 | 1521 | 38.1 | 5 | 20.1 | 0.64 | 225 | 7.2 | 89 |
| | | 2 | 8.7 | 0.4 | 676 | 36.3 | 13 | 18.3 | 0.49 | 596 | 5.8 | 31 |
| | | 3 | 7.2 | 0.25 | 465 | 16.3 | 15 | 14.5 | 0.35 | 235 | 5.7 | 62 |
| | | 4 | 6.6 | 0.25 | 1190 | 44.6 | 5 | 10.8 | 0.33 | 340 | 10.4 | 32 |
| | | 5 | 6.3 | 0.21 | 676 | 23.6 | 9 | 15.9 | 0.6 | 615 | 22.2 | 26 |
| | | 6 | 7.5 | 0.2 | 676 | 18.7 | 11 | 12.6 | 0.26 | 440 | 9.1 | 28 |
| | | 7 | 7.8 | 0.3 | 1593 | 54.1 | 5 | 24.6 | 1.1 | 597 | 25.8 | 41 |
| | | 8 | 7.2 | 0.4 | 549 | 31.0 | 12 | 11.4 | 0.6 | 348 | 17.6 | 33 |
| | | 9 | 7.8 | 0.37 | 771 | 36.7 | 10 | 14.4 | 0.5 | 996 | 36.1 | 14 |
| | | 10 | 8.4 | 0.4 | 1047 | 49.8 | 8 | 12.0 | 0.66 | 897 | 49.0 | 13 |
| | | Mean ± s.e.m. | 7.6 ± 0.2 | 0.29 ± 0.02 | 916 ± 126 | 35.0 ± 4.0 | 9 ± 1 | 15.4 ± 1.3 | 0.55 ± 0.07 | 528 ± 83 | 18.8 ± 4.6 | 37 ± 7 |
| Liver | Adenocarcinoma | 1 | 6.9 | 0.46 | 215 | 10.0 | 32 | 8.1 | 0.36 | 170 | 7.6 | 47 |
| | | 2 | 13.5 | 0.29 | 335 | 7.1 | 40 | 18.0 | 0.47 | 111 | 5.2 | 162 |
| | | 3 | 15.6 | 0.27 | 200 | 6.0 | 78 | 20.7 | 0.6 | 175 | 5.5 | 118 |
| | | 4 | 12.0 | 0.31 | 225 | 5.8 | 53 | 22.2 | 0.6 | 140 | 3.8 | 158 |
| | | 5 | 15.3 | 0.4 | 200 | 5.3 | 77 | 19.3 | 0.8 | 110 | 3.0 | 175 |
| | | 6 | 12.0 | 0.33 | 155 | 4.2 | 76 | 15.9 | 0.6 | 90 | 3.4 | 176 |
| | | Mean ± s.e.m. | 12.5 ± 1.2 | 0.34 ± 0.02 | 221 ± 24 | 6.4 ± 0.8 | 59 ± 8 | 17.3 ± 2.0 | 0.57 ± 0.06 | 132 ± 14 | 4.7 ± 0.6 | 139 ± 20 |

The activity is expressed as units per g wet tissue and as units per mg extracted protein. The comparisons for the U $mg^{-1}$ of protein data are as follows: PGM activity: normal lung vs normal liver; normal lung vs normal colon; normal liver vs normal colon, $P < 0.001$. Lung adenocarcinoma vs normal tissue, $P < 0.01$; lung squamous cell carcinoma vs normal tissue, $P < 0.04$; colon adenocarcinoma vs normal tissue, $P < 0.002$; hepatocarcinoma vs normal tissue, $P < 0.009$. BPGP activity: normal colon vs normal colon vs normal liver, $P < 0.001$. Lung adenocarcinoma vs normal tissue, $P < 0.01$; colon adenocarcinoma vs normal tissue, $P < 0.002$; hepatocarcinoma vs normal tissue, $P < 0.003$. All other comparisons are not significant.

Enolase molecules are dimers composed of three distinct subunits coded by separate genes and designated α (liver), β (muscle) and γ (brain) (for a review, see Day, 1982). The αα isoenzyme exists in most mammalian tissues. ββ and αβ enolase are found predominantly in skeletal and heart muscle. γγ and αγ enolases are present mainly in nervous tissue and in tissues with neuroendocrine cells. They have frequently been designated as neuron-specific enolase. The βγ hybrid has not been found, probably because the β and the γ enolase subunits are not expressed in the same tissue (for reviews, see Kato et al, 1983*a*; Taylor et al, 1983; Haimoto et al, 1985; Royds et al, 1985; Schmechel, 1985; Marangos and Schmechel, 1987).

The present study was undertaken to determine the distribution of total PGM, BPGP and enolase activities and isoenzymes in lung, colon and liver carcinoma as a first step to studying the expression of these isoenzymes in neoplastic cells. Only one report has been published about PGM isoenzymes in brain tumours (Omenn and Cheung, 1974; Omenn and Hermodson, 1975) and, to our knowledge, no data exist on other tumours. Numerous studies have been published on the distribution of enolase isoenzymes in human tumours (for reviews, see Taylor et al, 1983; Royds et al, 1985; Schmechel, 1985; Gerbitz et al, 1986; Marangos and Schmechel, 1987; Kaiser et al, 1989). However, most data have been obtained by immunohistochemical and immunoassay techniques. To our knowledge, only two reports have been published on the distribution of enolase isoenzyme proteins in lung tumours (Pahlman et al, 1986; Batandier et al, 1987), and no data exist on the distribution of enolase isoenzymes in colon and liver tumours.

### MATERIALS AND METHODS

### Materials

Enzymes, substrates, cofactors and biochemicals were purchased from either Boehringer (Mannheim, Germany) or Sigma (St Louis, MO, USA). β-Mercaptoethanol was from Merck (Darmstadt,

 © Cancer Research Campaign 1997

**Figure 1**: Electrophoretograms of PGM isoenzymes in extracts of human normal tissues and tumours. (**A**) Lung: lane 1, heart extract; lanes 3 and 5, squamous cell carcinoma; lane 7, adenocarcinoma; lanes 2, 4 and 6, normal tissues. (**B**) Colon: lanes 2, 4 and 6, adenocarcinoma; lanes 1, 3 and 5, normal tissues. (**C**) Liver: lanes 2, 4 and 6, adenocarcinoma; lanes 1, 3 and 5, normal tissues

**Table 2** PGM isoenzymes in lung, colon and liver normal tissues and tumours

| | | | Normal tissue | | | Tumour | | |
|---|---|---|---|---|---|---|---|---|
| **Tissue** | **Tumour** | **Case no.** | **MM** | **MB** | **BB** | **MM** | **MB** | **BB** |
| Lung | Adenocarcinoma | 1 | 0 | 0 | 100 | 0 | 0 | 100 |
| | | 2 | 0 | 8 | 92 | 0 | 8 | 92 |
| | | 3 | 0 | 0 | 100 | 0 | 0 | 100 |
| | | 4 | 0 | 0 | 100 | 0 | 0 | 100 |
| | | 5 | 0 | 3 | 97 | 0 | 0 | 100 |
| | | 6 | 0 | 3 | 97 | 0 | 8 | 92 |
| | | 7 | 0 | 0 | 100 | 0 | 0 | 100 |
| | Squamous cell carcinoma | 1 | 0 | 4 | 96 | 0 | 1 | 99 |
| | | 2 | 0 | 4 | 96 | 0 | 0 | 100 |
| | | 3 | 0 | 4 | 96 | 0 | 2 | 98 |
| | | 4 | 0 | 0 | 100 | 0 | 0 | 100 |
| | Carcinoid | 1 | 0 | 0 | 100 | 0 | 9 | 91 |
| | | 2 | 0 | 0 | 100 | 0 | 0 | 100 |
| Colon | Adenocarcinoma | 1 | 0 | 0 | 100 | 0 | 0 | 100 |
| | | 2 | 0 | 2 | 98 | 0 | 0 | 100 |
| | | 3 | 0 | 0 | 100 | 1 | 1 | 99 |
| | | 4 | 0 | 1 | 99 | 0 | 0 | 100 |
| | | 5 | 3 | 6 | 91 | 0 | 2 | 98 |
| | | 6 | 3 | 7 | 90 | 0 | 3 | 97 |
| | | 7 | 0 | 6 | 94 | 0 | 0 | 100 |
| | | 8 | 0 | 0 | 100 | 0 | 0 | 100 |
| | | 9 | 0 | 4 | 96 | 0 | 3 | 97 |
| | | 10 | 0 | 6 | 94 | 0 | 3 | 97 |
| Liver | Adenocarcinoma | 1 | 0 | 1 | 99 | 0 | 0 | 100 |
| | | 2 | | ND | | | ND | |
| | | 3 | 0 | 2 | 98 | 0 | 1 | 99 |
| | | 4 | 0 | 3 | 97 | 0 | 2 | 98 |
| | | 5 | 0 | 4 | 96 | 0 | 2 | 98 |
| | | 6 | 0 | 3 | 97 | 0 | 0 | 100 |

The results are expressed as a percentage of the total activity on electrophoresis. ND, not determined.

Germany) and bovine serum albumin was from Calbiochem (La Jolla, CA, USA). Other chemicals were reagent grade. Agar noble was obtained from Difco Laboratories (Detroit, MI, USA). Cellulose acetate strips were from Helena Laboratories (Beaumont, TX, USA) and agarose gels were from Ciba-Corning (Palo Alto, CA, USA).

### Tissue samples

Tumour samples were obtained from surgical resection specimens: seven lung adenocarcinomas, five lung squamous cell carcinomas, two carcinoid tumours of the lung, ten colon adenocarcinomas and six hepatocarcinomas. Samples of normal tissue were obtained

© Cancer Research Campaign 1997

from adjacent normal tissue that had to be removed during tumour surgery.

### Tissue extraction

Tissue extracts were prepared by homogenization in three volumes (w/v) of cold 20 mM Tris-HCl buffer, pH 7.5, containing 1 mM EDTA and 1 mM β-mercaptoethanol with a Polytron homogenizer (Lucerne, Switzerland) (position 5, 20 s). Cellular debris was removed by centrifugation at 4°C for 30 min at 12 500 ***g***, and the supernatants were used for the assay of enzyme activities and isoenzymes.

### Enzyme and protein assays

PGM activity was measured spectrophotometrically at 30°C by coupling the formation of 2-phosphoglycerate from 3-phosphoglycerate with the enolase, pyruvate kinase and lactate dehydrogenase catalysed reactions (Beutler and Stratton, 1975), as previously described (Durany and Carreras, 1996). BPGP activity was assayed by measuring the appearance of inorganic phosphate from 2,3-bisphosphoglycerate (Joyce and Grisolia, 1958). The reaction mixture was contained in a total volume of 1 ml: 50 mM triethanolamine, pH 7.6, 1 mM 2,3-bisphosphoglycerate, 2.5 mM 2-phosphoglycolate (as activator) and the sample tested. After incubation at 37°C, the reaction was stopped by the addition of 0.25 ml of 25% trichloroacetic acid. After centrifugation, the inorganic phosphate was estimated in the supernatant by the method of Itaya and Ui (1966). Enolase activity was measured spectrophotometrically at 30°C by coupling the formation of phosphoenolpyruvate with the pyruvate kinase and lactate dehydrogenase catalysed reactions (Bergmeyer, 1983), as previously described (Joseph et al, 1996). Enzyme activities were expressed as U $g^{-1}$ wet tissue and as U $mg^{-1}$ protein (1 Unit = 1 μmol substrate converted per min). Protein was determined by the method of Bradford (1976), using bovine serum albumin as a standard.

### Isoenzyme analysis

The methods described previously were used to evaluate PGM isoenzymes by cellulose acetate electrophoresis (Durany and Carreras, 1996) and enolase isoenzymes by agarose gel electrophoresis (Joseph et al, 1996).

### Separation of BPGP from PGM and BPGM

PGM isoenzymes, BPGM and the BPGM-PGM hybrids were separated from 2-phosphoglycolate non-stimulated BPGP by highly resolutive gel filtration fast liquid chromatography (FPLC system and Superdex 75 HR column from Pharmacia LKB Biotechnology, Sweden). The column was equilibrated with extraction buffer. A sample (0.2 ml) of the tissue extract was injected, the column was eluted at a flow rate of 0.1 ml $min^{-1}$ and 500-μl fractions were collected.

### Statistical analysis

A one-way analysis of variance with repeated measures was employed for statistical evaluation and used to compare enzyme activities among different tissues. When a significant *P*-value was obtained ($P < 0.05$), the difference between means was located with the Tukey test (Baylar and Mosteller, 1992). To compare PGM activity in tumour and control tissues, the Wilcoxon *t*-test was used (Baylar and Mosteller, 1992). Values are reported as means ± s.e.m. Data were analysed by InStat statistical software.

### RESULTS

### Distribution of PGM and BPGP activities and isoenzymes

Table 1 summarizes the levels of total PGM and BPGP activities in normal lung, colon and liver tissues, and in their tumours. Figure 1 shows some of the PGM isoenzyme patterns determined by cellulose acetate electrophoresis, and Table 2 summarizes the distribution of PGM isoenzymes in normal and tumour tissues.

As shown, lung tissue presents a lower PGM content than liver and colon tissues ($P < 0.001$), which have similar PGM concentration. Normal lung, liver and colon tissues contain almost exclusively BB-PGM with traces of MB-PGM isoenzyme. Colon is the tissue with the highest BPGP activity ($P < 0.001$), and no significative difference is observed between the levels of this activity in lung and liver. The PGM–BPGP activity ratio in colon and in lung is not significantly different. In contrast, liver possesses a higher PGM–BPGP activity ratio. These results indicate that lung, colon and liver tissues differ in their PGM content and in the concentration of some of the other enzymes that also possess BPGP activity (BPGM, BPGM–PGM hybrid and 2-phosphoglycolate non-stimulated BPGP).

It has been shown that 2-phosphoglycolate non-stimulated BPGP has a lower molecular weight than PGM, BPGM and BPGM–PGM hybrids, and that they can be separated by gel filtration chromatography (Carreras et al, 1981; Pons et al, 1985*b*). Therefore, we compared the PGM–BPGP activity ratio in liver and lung extracts before and after gel filtration FPLC. It was found that neither the enzyme activities nor the PGM–BPGP activity ratio changed significantly after chromatography. There was correlation between the levels of the enzyme activities in the crude extracts and in the peaks containing the PGM, the BPGM and the BPGM–PGM hybrid isolated by gel filtration chromatography. The peak from liver extract was the peak with the highest PGM activity. It was concluded that the contribution of the 2-phosphoglycolate non-stimulated BPGP enzyme to the total BPGP activity of the tissues is almost negligible, and that they differ in the concentration of PGM, BPGM and BPGM–PGM hybrid.

As summarized in Table 1, all tumours present higher PGM activity levels than the corresponding normal tissues. However, no changes are observed in the PGM isoenzyme patterns. As the normal tissues, tumours contain mainly type BB-PGM, with only traces of the MB-PGM form (Figure 1 and Table 2). In contrast to PGM activity, the levels of BPGP activity in all tumours are lower than in normal tissues. Correspondingly, the PGM–BPGP activity ratios in tumours are higher than in control tissues.

The fact that tumours present opposite changes in the levels of PGM activity and of BPGP activity suggests that tumoral tissues change both the concentration of PGM and the concentration of the other enzymes that also have BPGP activity. Extracts of liver carcinoma and of lung carcinoid were filtered through a FPLC column in order to separate the 2-phosphoglycolate non-stimulated BPGP. The total enzyme activities and the PGM–BPGP activity ratio of the extracts were similar before and after chromatography. This shows that, as in normal tissues, the contribution of 2-phosphogly-

© Cancer Research Campaign 1997

**Table 3** Levels of enolase activity in human lung, colon, and liver normal tissues and tumours

| Tissue | Tumour | Case no. | | Normal tissue U g$^{-1}$ | Normal tissue U mg$^{-1}$ | Tumour U g$^{-1}$ | Tumour U mg$^{-1}$ |
|---|---|---|---|---|---|---|---|
| Lung | Adenocarcinoma | 1 | | 6 | 0.1 | 17 | 0.3 |
| | | 2 | | 5 | 0.07 | 21 | 0.3 |
| | | 3 | | 3 | 0.1 | 20 | 0.5 |
| | | 4 | | 2 | 0.08 | 26 | 0.86 |
| | | 5 | | 5 | 0.12 | 17 | 0.47 |
| | | 6 | | 7 | 0.15 | 19 | 0.48 |
| | | 7 | | 4 | 0.12 | 11 | 0.28 |
| | | | Mean ± s.e.m. | 4.5 ± 0.6 | 0.10 ± 0.01 | 18.7 ± 1.7 | 0.45 ± 0.07 |
| | Squamous cell Carcinoma | 1 | | 15 | 0.2 | 23 | 0.2 |
| | | 2 | | 9 | 0.1 | 24 | 0.5 |
| | | 3 | | 3 | 0.06 | 16 | 0.3 |
| | | 4 | | 8 | 0.12 | 33 | 0.61 |
| | | 5 | | 4 | 0.17 | 7 | 0.36 |
| | | | Mean ± s.e.m. | 7.9 ± 2.1 | 0.13 ± 0.02 | 20.6 ± 4.3 | 0.39 ± 0.07 |
| | Carcinoid | 1 | | 3 | 0.1 | 5 | 0.23 |
| | | 2 | | 4 | 0.07 | 11 | 0.19 |
| | | | Mean ± s.e.m. | 3.5 ± 0.5 | 0.085 ± 0.01 | 8.0 ± 3.0 | 0.21 ± 0.02 |
| Colon | Adenocarcinoma | 1 | | 6.1 | 0.1 | 19.6 | 0.4 |
| | | 2 | | 7.0 | 0.1 | 8.3 | 0.1 |
| | | 3 | | 5.4 | 0.1 | 14.5 | 0.2 |
| | | 4 | | 5.2 | 0.09 | 14.3 | 0.2 |
| | | 5 | | 5.1 | 0.2 | 17.8 | 0.4 |
| | | 6 | | 7.6 | 0.2 | 8.7 | 0.1 |
| | | 7 | | 6.9 | 0.1 | 12.9 | 0.3 |
| | | 8 | | 4.5 | 0.2 | 11.2 | 0.3 |
| | | 9 | | 7.4 | 0.1 | 18.2 | 0.4 |
| | | 10 | | 8.5 | 0.1 | 11.6 | 0.2 |
| | | | Mean ± s.e.m. | 6.4 ± 0.4 | 0.13 ± 0.015 | 13.7 ± 1.2 | 0.26 ± 0.04 |
| Liver | Hepatocarcinoma | 1 | | 9 | 0.8 | 8 | 0.5 |
| | | 2 | | 14 | 0.2 | 22 | 0.4 |
| | | 3 | | 15 | 0.2 | 21 | 0.6 |
| | | 4 | | 11 | 0.2 | 12 | 0.3 |
| | | 5 | | 17 | 0.4 | 20 | 0.8 |
| | | 6 | | 18 | 0.3 | 20 | 0.7 |
| | | | Mean ± s.e.m. | 14 ± 1.4 | 0.35 ± 0.1 | 17.2 ± 2.3 | 0.56 ± 0.07 |

The activity is expressed as units per g wet tissue and as units per mg extracted protein. The comparisons for the U mg$^{-1}$ of protein data are as follows: normal lung vs normal liver, $P$<0.001; normal colon vs normal liver, $P$<0.01; lung adenocarcinoma vs normal tissue, $P < 0.05$, colon adenocarcinoma vs normal tissue, $P < 0.05$. All other comparisons are not significant.

**Figure 2** Electrophoretograms of enolase isoenzymes in extracts of human lung normal tissue and tumours. (**A**) Adenocarcinoma: lanes 2, 4 and 6, tumours; lanes 3, 5 and 7, normal tissues. (**B**) Squamous cell carcinoma: lanes 8 and 10, tumours; lanes 9 and 11, normal tissues. (**C**) Carcinoid: lanes 12 and 14, tumours; lanes 13 and 15, normal tissues. Lanes 1 and 16, human brain extracts (cortex)

© Cancer Research Campaign 1997 

**Figure 3**: Electrophoretograms of enolase isoenzymes in extracts of human liver and colon normal tissues and tumours. (**A**) Liver: lanes 2, 4 and 6, hepatocarcinomas; lanes 3, 5 and 7, normal tissues. (**B**) Colon: lanes 9, 11 and 13, adenocarcinomas; lanes 10, 12 and 14, normal tissues. Lanes 1 and 8 human brain extracts (cortex)

**Table 4** Enolase isoenzymes in human lung, colon and liver normal tissues and tumours

| Tissue | Tumour | Case No. | Normal tissue αα[a] | Normal tissue αγ[b] | Normal tissue γγ[c] | Tumour αα | Tumour αγ | Tumour γγ |
|---|---|---|---|---|---|---|---|---|
| Lung | Adenocarcinoma | 1 | 77 | 20 | 3 | 94 | 4 | 2 |
| | | 2 | 62 | 31 | 7 | 85 | 13 | 2 |
| | | 3 | 73 | 23 | 4 | 76 | 22 | 2 |
| | | 4 | 73 | 23 | 4 | 85 | 13 | 2 |
| | | 5 | 77 | 19 | 4 | 84 | 14 | 2 |
| | | 6 | 76 | 21 | 3 | 96 | 4 | 0 |
| | | 7 | 76 | 22 | 2 | 78 | 20 | 2 |
| | | Mean ± s.e.m. | 73.4 ± 2.0 | 22.7 ± 1.5 | 3.8 ± 0.6 | 85.4 ± 2.8 | 12.8 ± 2.6 | 1.7 ± 0.3 |
| | Squamous cell carcinoma | 1 | 74 | 22 | 4 | 84 | 15 | 1 |
| | | 2 | 90 | 10 | 0 | 92 | 8 | 0 |
| | | 3 | 81 | 17 | 2 | 85 | 13 | 2 |
| | | 4 | 84 | 14 | 2 | 70 | 28 | 2 |
| | | 5 | 78 | 20 | 2 | 88 | 12 | 0 |
| | | Mean ± s.e.m. | 81.4 ± 2.7 | 16.6 ± 2.1 | 2.0 ± 0.6 | 83.8 ± 3.7 | 15.2 ± 3.4 | 1.0 ± 0.4 |
| | Carcinoid | 1 | 70 | 26 | 4 | 44 | 32 | 24 |
| | | 2 | 70 | 25 | 5 | 50 | 37 | 13 |
| | | Mean ± s.e.m. | 70 ± 0.0 | 25.5 ± 0.5 | 4.5 ± 0.5 | 47 ± 3 | 34.5 ± 2.5 | 18.5 ± 5.5 |
| Colon | Adenocarcinoma | 1 | 77 | 19 | 4 | 86 | 12 | 2 |
| | | 2 | 70 | 24 | 6 | 87 | 10 | 3 |
| | | 3 | 70 | 21 | 9 | 89 | 9 | 2 |
| | | 4 | 74 | 20 | 6 | 84 | 14 | 2 |
| | | 5 | 75 | 19 | 6 | 90 | 8 | 2 |
| | | 6 | 79 | 15 | 6 | 94 | 4 | 2 |
| | | 7 | 69 | 25 | 6 | 79 | 19 | 2 |
| | | 8 | 89 | 8 | 3 | 82 | 15 | 3 |
| | | 9 | 84 | 12 | 4 | 86 | 11 | 3 |
| | | 10 | 77 | 18 | 5 | 81 | 16 | 3 |
| | | Mean ± s.e.m. | 76.4 ± 2.0 | 18.1 ± 1.6 | 5.5 ± 0.5 | 85.8 ± 1.4 | 11.8 ± 1.4 | 2.4 ± 0.2 |
| Liver | Hepatocarcinoma | 1 | 96 | 3 | 1 | 93 | 6 | 1 |
| | | 2 | 93 | 6 | 1 | 94 | 4 | 2 |
| | | 3 | 92 | 6 | 2 | 100 | 0 | 0 |
| | | 4 | 95 | 5 | 0 | 96 | 3 | 1 |
| | | 5 | 95 | 5 | 0 | 97 | 3 | 0 |
| | | 6 | 94 | 4 | 2 | 100 | 0 | 0 |
| | | Mean ± s.e.m. | 94.2 ± 0.6 | 4.8 ± 0.5 | 1.0 ± 0.3 | 96.7 ± 1.2 | 2.6 ± 0.9 | 0.6 ± 0.3 |

The results are expressed as a percentage of the total enolase activity on electrophoresis. The comparisons are as follows: [a]Lung vs liver, $P < 0.001$; colon vs liver, $P < 0.001$. [b]Lung vs liver, $P < 0.001$; colon vs liver, $P<0.001$. [c]Lung vs colon, $P < 0.01$; lung vs liver, $P < 0.05$; colon vs liver, $P < 0.001$. All other comparisons are not significant.

 © Cancer Research Campaign 1997

colate non-stimulated BPGP to the total BPGP activity in tumour tissues is not relevant. In addition, these results indicate that the decrease of the total BPGP activity found in tumours is caused by a decrease of the BPGM and of the BPGM–PGM hybrid. It has to be noted that there was a correlation between the assay of the enzyme activities in the crude extracts and in the peaks containing the PGM, the BPGM and the BPGM–PGM hybrid isolated by gel filtration chromatography. Those peaks corresponding to tumour tissues had higher PGM activity and lower BPGP activity than the peaks corresponding to normal tissues.

### Distribution of enolase activity and isoenzymes

Table 3 summarizes the levels of total enolase activity in normal human lung, colon and liver tissues, and in their tumours. Figures 2 and 3 show the electrophoretograms of some tumours determined by agarose gel electrophoresis, and Table 4 summarizes the data obtained.

As shown, liver tissue possesses a higher enolase content than lung ($P < 0.001$) and colon ($P < 0.01$) tissues, which have similar enolase concentration. All tumours have higher enolase activity than the corresponding normal tissues, although only in lung and colon adenocarcinoma are the differences observed statistically significant.

The normal tissues possess all the three enolase isoenzymes, although the proportion of αα-enolase is much larger than the proportions of αγ-and γγ-enolase. Normal lung and colon tissues present enolase isoenzymes in similar proportions. Liver tissue has lower proportions of enolase isoenzymes containing the γ subunit (αγ- and γγ-enolase). Five of seven lung adenocarcinomas and four of five squamous cell carcinomas of the lung show a lower content of αγ- and γγ-enolase than the corresponding normal tissue. In contrast, one squamous cell carcinoma and two carcinoid tumours of the lung present higher proportions of the enolase isoenzymes containing the γ subunit than the normal tissues. Colon and liver carcinomas have lower proportions of αγ- and γγ-enolase than normal tissues, although in hepatocarcinomas the decrease in the proportion of the enolase isoenzymes containing the γ subunit is less evident than in colon adenocarcinomas.

### DISCUSSION

Our results show that, in normal human lung, colon and liver tissue, the PGM and the enolase activities are much higher than the BPGP activity, which is in agreement with the different functions of these enzymes. PGM and enolase are enzymes of the main glycolytic pathway, whereas the BPGP activity participates in the 2,3-bisphosphoglycerate bypass (or Rapoport–Luebering shunt), a collateral deviation of glycolysis (Rapoport, 1968).

The data presented on the distribution of PGM isoenzymes in normal human lung, colon and liver agree with preliminary results (Durany and Carreras, 1996) and confirm that in all these tissues type BB-PGM is the predominant PGM isoenzyme. They contain traces of MB-PGM and no detectable MM-PGM. Our results also show that in these tissues most of the total BPGP activity is caused by PGM, BPGM and BPGM-PGM hybrid. The contribution of 2-phosphoglycolate non-stimulated BPGP is very low. Similar results were obtained in other tissues of pig and cat (Carreras et al, 1981; Pons et al, 1985*b*).

Our data on the distribution of enolase isoenzymes in tissues are in agreement with the results of others. It has been shown by immunoassay that the three enolase subunits are present in normal human lung, colon and liver, although the proportion of α subunit is much larger than the proportions of β and γ subunits (Schmechel et al, 1978; Marangos et al, 1979, 1980; Kato et al, 1983*a*, *b*; Taylor et al, 1983; Fujita et al, 1987; Marangos and Schmechel, 1987). Hullin et al (1980) found that γγ-enolase determined by ion-exchange chromatography represented 0.7%, 1.3% and 0.04% of the total enolase activity in extracts of lung, large intestine and liver respectively. Batandier et al (1987) showed by electrophoretic analysis that αα-enolase was the predominant isoenzyme in human lung. αγ-Enolase represented a low proportion ($5.75 \pm 0.25\%$) of the total enolase activity and γγ-enolase was not detectable. The results presented here confirm that the γ-enolase subunit is expressed in normal lung, colon and liver, and show that, in lung and in colon tissues, αγ-enolase represents an appreciable proportion ($21 \pm 5.1\%$ and $18.1 \pm 1.6\%$ respectively) of the total enolase activity. In liver, the proportion of αγ-enolase is lower ($4.8 \pm 0.5\%$) than in the two other tissues.

In all lung, colon and liver tumours, we found an increase in PGM and enolase activities and a decrease in 2-phosphoglycolate-stimulated BPGP activity. In contrast, in brain tumours a decrease of the enolase and PGM activities was observed (Joseph et al, 1996; N Durany and J Carreras, unpublished results).

In the tumours studied, we have not detected changes in the distribution of PGM isoenzymes, but we have observed some alterations in the enolase isoenzyme patterns. In colon and liver carcinomas, and in most adenocarcinomas and squamous cell carcinomas of the lung, we found a decrease in enolase isoenzymes containing type γ subunit (αγ- and γγ-enolase). In contrast, in two carcinoid tumours and in one squamous cell carcinoma of the lung, we observed an increase in these enolase isoenzymes. In addition, we have deduced that the decrease of the 2-phosphoglycolate-stimulated BPGP activity observed in all tumours reflects a decrease of the BPGM and the BPGM–PGM hybrid enzyme levels.

Our results on enolase isoenzymes in tumours can be compared with data from other authors but, to our knowledge, no previous data exist on PGM isoenzymes in lung, colon and liver tumours. By enzyme immunoassay, it has been shown that the α-enolase subunit is abundant and that the β-enolase subunit is present at low levels in most lung tumours (Nakajima et al, 1985; Fujita et al, 1987). The presence of the γ-enolase subunit in lung neuroendocrine tumours has been well documented by immunohistochemistry, but its presence in non-neuroendocrine tumours is controversial (for reviews, see Marangos and Schmechel, 1987; Kaiser et al, 1989). Pahlman et al (1986) found using ion-exchange chromatography that non-small-cell carcinoma cell lines had measurable quantities of neuron-specific enolase, although its levels were 10- to 100-fold lower than those found in small-cell carcinoma cell lines. Batandier et al (1987) found by electrophoresis αγ-enolase in significantly higher proportion in lung neuroendocrine carcinomas than in non-neuroendocrine tumours. γγ-Enolase was present at high levels in lung neuroendocrine carcinomas and consistently absent in non-neuroendocrine tumours. In disagreement with these authors, we have detected γγ-enolase in non-neuroendocrine lung tumours, although at lower levels than in normal lung tissues and lung carcinoids. The discrepancy is probably owing to a higher sensitivity of the method of detection used by us.

By immunoassay, the γ-enolase subunit was detected in gastric and gut carcinoids (Tapia et al, 1981; Simpson et al, 1984; Nash et al, 1986), and in a cell line from a neuroendocrine tumour of colon (Reeve et al, 1986). In contrast, no γ-enolase staining was found in colon adenocarcinomas (Simpson et al, 1984; Vinores et al, 1984; Fukuda et al, 1989), and low levels of reactivity were detected in a cell line from a colon carcinoma (Reeve et al, 1986). As indicated above, we have detected αγ- and γγ-enolase in colon adenocarcinomas, although at a lower proportion than in normal tissue.

By immunohistochemistry, γ-enolase was found to be negative in hepatocellular carcinoma (Vinores et al, 1984; Fujita et al, 1987), although it was present in one of two cases of fibrolamellar carcinoma with neurosecretory granules (Fujita et al, 1987). Our results indicate that, although at a very low proportion, αγ- and γγ-enolase isoenzymes can be detected in liver carcinomas.

In conclusion, we have found that in hepatocarcinoma, colon and lung adenocarcinoma, lung squamous cell carcinoma and carcinoid tumour of the lung, the expression of PGM subunits does not suffer any qualitative change. In tumour tissues, the total PGM activity increases but the relative expression of type B and type M PGM subunits does not vary. As in the normal tissues, type B subunit is predominant. The total enolase activity also increases in all tumours studied by us but, in contrast to PGM, the distribution of enolase isoenzymes in tumour tissues presents some alterations. In two carcinoid tumours and in one squamous cell carcinoma of the lung, the proportion of enolase isoenzymes containing type γ subunit was found to be increased. In all other tumours, it was found to be decreased. The changes in the proportions of enolase isoenzymes in tumours could reflect a change in the proportions of the different cell types present in the tissues. But a change in the expression of α- and γ-enolase subunits cannot be excluded, since it has been shown that cells undergoing mitosis activate the expression of the α-enolase subunit. It has been reported that α-enolase is expressed at relatively high levels in all actively proliferating human cell lines but at very low levels in normal resting peripheral blood lymphocytes, in which its synthesis is induced upon mitogenic stimulation (Giallongo et al, 1986*a*, *b*). Moreover, it has been shown that expression of the genes encoding four glycolytic enzymes, including enolase, is specifically stimulated in quiescent rat fibroblast by either epidermal growth factor or serum (Matrisian et al, 1985).

From the diagnostic point of view, we conclude that PGM and enolase isoenzymes are not good markers for the tumours studied by us. Only in carcinoid tumours of the lung can the increase in neuron-specific enolase have clinical applications, as already indicated by others (Royds et al, 1985; Schemechel, 1985; Gerbitz et al, 1986; Marangos and Schmechel, 1987; Kaiser et al, 1989).

### ABBREVIATIONS

BPGM, 2,3-bisphosphoglycerate mutase; BPGP, 2,3-bisphosphoglycerate phosphatase; PGM, phosphoglycerate mutase.

### ACKNOWLEDGEMENTS

This work was supported by FIS, grant no.93/0573, by Generalitat de Catalunya, grant GRQ 94-1036, and by Hospital Clinic de Barcelona, grant 1993. We are grateful to J Ojuel and J Parra for advice on the statistical analysis.

### REFERENCES

Adamson ED (1976) Enzyme transitions of creatine phosphokinase, aldolase and phosphoglycerate mutase in differentiating mouse cells. *J Embryol Exp Morphol* **35**: 355–367

Bartrons R and Carreras J (1982) Purification and characterization of phosphoglycerate mutase isozymes from pig heart. *Biochim Biophys Acta* **708**: 167–177

Batandier C, Brambilla E, Jacrot M, Morel F, Beriel H, Paramelle B and Brambilla C (1987) Isoenzyme pattern of enolase in the diagnosis of neuroendocrine bronchopulmonary tumors. *Cancer* **60**: 838–843

Baylar III JC and Mosteller F (eds) (1992) *Medical Uses of Statistics* 2nd edn, pp. 246–250. NEJM Books: Boston

Bergmeyer HU and Graβl M (1983) Enolase. In *Methods of Enzymatic Analysis* Vol.2 Bergmeyer J and Graβl M (eds), pp. 182–183. Verlag Chemie Press: Weinheim

Beutler E (ed.) (1975) Monophosphoglyceromutase (MPGM). In *Red Cell Metabolism* pp. 56–58. Grune & Stratton: New York

Bradford M (1976) A rapid and sensitive method for the quantification of microgram quantities of protein utilizing the principle of protein-dye binding. *Anal Biochem* **72**: 248–254

Carreras J, Bartrons R, Bosch J and Pons G (1981) Metabolism of glycerate-2,3-$P_2$-1. Distribution of the enzymes involved in the glycerate-2,3-$P_2$ metabolism in pig tissues. *Comp Biochem Physiol* **70B**: 477–485

Day INM (1982) Enolases and PGP 9.5 as tissue-specific markers. *Biochem Soc Trans* **20**: 637–642

Durany N and Carreras J (1966) Distribution of phosphoglycerate mutase isozymes in rat, rabbit and human tissues. *Comp Biochem Physiol* **114B**: 217–223

Edwards YH and Hopkinson DA (1977) Developmental changes in the electrophoretic patterns of human enzymes and other proteins. In *Isozymes: Current Topics in Biological and Medical Research*, Ratazzi MC, Scandalios JG and Whit GS (eds), pp. 19–78. 19–78. Alan R. Liss Inc: New York

Fothergill-Gilmore LA and Watson HC (1989) The phosphoglycerate mutases. *Adv Enzymol* **62**: 227–313

Fujita K, Haimoto H, Imaizumi M, Abe T and Kato K (1987) Evaluation of γ-enolase as a tumor marker for lung cancer. *Cancer* **60**: 362–369

Fukuda Y, Miyazawa Y, Imoto M, Koyama Y, Nakano I, Nagura H and Kato K (1989) *In situ* distribution of enolase isozymes in chronic liver disease. *Am J Gastroenterol* **84**: 601–605

Fundele R, Winking H, Illmensee K and Jägerbauer E-M (1987) Developmental activation of phosphoglycerate mutase-2 in the testis of the mouse. *Devel Biol* **124**: 562–566

Gerbitz KD, Summer J and Schumacher I (1986) Enolase isoenzymes as tumour markers. *J Clin Chem Clin Biochem* **24**: 1009–1016

Giallongo A, Feo S, Showe LC and Croce CM (1986*a*) Isolation and partial characterization of a 48-kDa protein which is induced in normal lymphocytes upon mitogenic stimulation. *Biochem Biophys Res Commun* **134**: 1238–1244

Giallongo A, Feo S, Moore R, Croce CM and Showe LC (1986*b*) Molecular cloning and nucleotide sequence of a full-length cDNA for human α enolase. *Proc Natl Acad Sci USA* **83**: 6741–6745

Haimoto H, Takahashi Y, Koshikawa T, Nagura H and Kato K (1985) Immunohistochemical localization of γ-enolase in normal human tissues other than nervous and neuroendocrine tissues. *Lab Invest* **52**: 257–263

Hullin DA, Brown K, Kynoch PAM, Smith CH and Thompson RJ (1980) Purification, radioimmunoassay and distribution of human brain 14-3-2 protein (nervous-system specific enolase) in human tissues. *Biochim Biophys Acta* **628**: 98–108

Itaya K and Ui M (1966) A new micromethod for the colorimetric determination of inorganic phosphate. *Clin Chim Acta* **14**: 361–366

Joseph J, Cruz-Sánchez FF and Carreras J (1996) Enolase activity and isoenzyme distribution in human brain regions and tumors. *J Neurochem* **66**: 2484–2490

Joyce BK and Grisolia S (1958) Studies on glycerate-2,3-diphosphatase. *J Biol Chem* **233**: 350–354

Kaiser E, Kuzmits R, Pregant P, Burghuber O and Worofka W (1989) Clinical biochemstry of neuron specific enolase. *Clin Chim Acta* **183**: 13–32

Kappel W and Hass LF (1976) The isolation and partial characterization of diphosphoglycerate mutase from human erythrocytes. *Biochemistry* **15**: 290–295

Kato K, Ishiguro Y and Ariyoshi Y (1983*a*) Enolase isozymes as disease markers: distribution of three enolase subunits (α, β, and γ) in various human tissues. *Disease Markers* **1**: 213–220

Kato K, Okagawa Y, Suzuki F, Shimizu, Mokuno K and Takahashi Y (1983*b*) Immunoassay of human muscle enolase subunit in serum: a novel marker antigen for muscle diseases. *Clin Chim Acta* **131**: 75–85

Marangos PJ and Schmechel DE (1987) Neuron specific enolase, a clinically useful marker for neurons and nonendocrine cells. *Annu Rev Neurosci* **10**: 269–95

Marangos PJ, Schmechel D, Parma AM, Clark RL and Goodwin FK (1979) Measurement of neuron-specific (NSE) and non-neuronal (NNE) isoenzymes of enolase in rat, monkey and human nervous tissue. *J Neurochem* **33**: 319–329

 © Cancer Research Campaign 1997

Marangos PJ, Campbell IC, Schmechel DE, Murphy DL and Goodwin FK (1980) Blood platelets contain a neuron-specific enolase subunit. *J Neurochem* **34(5)**: 1254–1258

Matrisian LM, Rautmann G, Magun BE and Breathnach R (1985) Epidermal growth factor or serum stimulation of rat fibroblasts induces an elevation in mRNA levels for lactate dehydrogenase and other glycolitic enzymes. *Nucleic Acids Res* **13**: 711–726

Mezquita J and Carreras J (1981) Phylogeny and ontogeny of the phosphoglycerate mutases I. Electrophoretic phenotypes of the glycerate-2,3-$P_2$ dependent phosphoglycerate mutase in vertebrates. *Comp Biochem Physiol* **70B**: 237–245

Mezquita J, Bartrons R, Pons G and Carreras J (1981) Phylogeny and ontogeny of the phosphoglycerate mutases II. Characterization of phosphoglycerate mutase isozymes from vertebrates by their thermal lability and sensitivity to the sulfhydryl group reagents. *Comp Biochem Physiol* **70B**: 247–255

Nakajima T, Kato K, Tsumuraya M, Kodama T, Shimosato Y and Kameya T (1985) The levels of three enolase subunits in human tumors: a low α-/γ-subunit ratio as indicator of tumors of neuronal and neuroendocrine nature. *Neurochem Int* **7**: 615–619

Narita H, Utsumi S, Ikura K, Sasaki R and Chiba H (1979) Comparative studies of the enzymes involved in 2,3-bisphosphoglycerate metabolism of rabbit erythrocytes and muscle cells. *Int J Biochem* **10**: 25–38

Nash SV and Said JW (1986) A histochemical and immunohistochemical study of epithelial (keratin proteins, carcinoembryonic antigen) and neuroendocrine (neuron-specific enolase, bombesin and chromogranin) markers in foregut, midgut, and hindgut tumors. *J Clin Pathol* **86**: 415–422

Omenn GS and Cheung C-Y (1974) Phosphoglycerate mutase isozyme marker for tissue differentiation in man. *Am J Hum Genet* **26**: 393–399

Omenn GS and Hermodson MA (1975) Human phosphoglycerate mutase: isozyme marker for muscle differentiation and for neoplasia. In *Isozymes* Vol. 3, Markert CL (ed.), pp. 1005–1019. Academic Press: New York

Pahlman S, Esscher T and Nilsson K (1986) Expression of γ-subunit of enolase, neuron-specific enolase, in human non-neuroendocrine tumors and derived cell lines. *Lab Invest* **54**: 554–560

Pons G, Bartrons R and Carreras J (1985*a*) Hybrid forms of phosphoglycerate mutase and 2,3-bisphosphoglycerate synthase-phosphatase. *Biochem Biophys Res Commun* **129**: 658–663

Pons G, Berrocal F, Tauler A and Carreras J (1985*b*) Metabolism of glycerate-2,3-$P_2$-VII. Enzymes involved in the metabolism of glycerate-2,3-$P_2$ in cat tissues. *Comp Biochem Physiol* **80B**: 551–556

Prehu MO, Calvin MC, Prehu C and Rosa R (1984) Biochemical and immunological arguments for homology between red cell and liver phosphoglyceromutase isozymes. *Biochim Biophys Acta* **787**: 270–274

Rapoport S (1968) The regulation of glycolysis in mammalian erythrocytes. *Essays Biochem* **4**: 69–103

Reeve JG, Stewart J, Watson JV, Wulfrank D, Twentyman PR and Bleehen NM (1986) Neuron specific enolase expression in carcinoma of the lung. *Br J Cancer* **53**: 519–528

Rosa R, Audit I and Rosa J (1975) Presence of a diphosphoglycerate phosphatase activity in the multiple molecular forms of 3-phosphoglycerate mutase. In *Isozymes* Vol.3, Market CL (ed.), pp. 695–706. Academic Press: New York

Rosa R, Calvin M-C, Préhu M-O, Levy-Strauss M and Rosa J (1984) Evidence for the presence of a hybrid phosphoglyceromutase/bisphosphoglyceromutase in the red cells: partial characterization of the hybrid. *Biochem Biophys Res Commun* **120**: 715–720

Royds JA, Taylor CB and Timperley WR (1985) Enolase isoenzymes as diagnostic markers. *Neuropathol Appl Neurobiol* **11**: 1–16

Sasaki R, Ikura K, Narita H and Chiba H (1976) Multifunctionality of the enzyme involved in the 2,3-disphosphoglycerate metabolism of pig erythrocytes. *Agric Biol Chem* **40**: 2213–2224

Sasaki R, Ikura K, Narita H and Chiba H (1975) Purification of bisphosphoglyceromutase, 2,3-bisphosphoglycerate phosphatase and phosphoglyceromutase from human erythrocytes. *Eur J Biochem* **50**: 581–593

Schmechel D (1985) γ-Subunit of the glycolitic enzyme enolase: nonspecific or neuron specific? *Lab Invest* **52**: 239–242

Schmechel D, Marangos PJ and Brightman M (1978) Neuron-specific enolase is a molecular marker for peripheral and central neuroendocrine cells. *Nature* **276**: 834–836

Simpson S, Vinik AI, Marangos PJ and Lloyd RV (1984) Immunohistochemical localization of neuron-specific enolase in gastroenteropancreatic neuroendocrine tumors. *Cancer* **54**: 1364–1369

Tapia FJ, Barbosa AJA, Marangos PJ, Polak JM, Bloom SR, Dermody C and Pearse AGE (1981) Neuron-specific enolase is produced by neuroendocrine tumors. *Lancet* **1**: 808–811

Taylor CB, Royds JA, Parsons MA and Timperley WR (1983) Diagnostic aspects of enolase isozymes. *Curr Topics Biol Med Res* **11**: 95–119

Vinores SA, Bonnin JM, Rubinstein LJ and Marangos PJ (1984) Immunohistochemical demonstration of neuron-specific enolase in neoplasms of the CNS and other tissues. *Arch Pathol Lab Med* **108**: 536–540

Wold F (1971) Enolase. In *The Enzymes* 3rd edn, vol. V, Boyer PD (ed.), pp. 499–538. Academic Press: New York

© Cancer Research Campaign 1997